# Impaction caused by a rare erupted peripheral compound odontoma

**DOI:** 10.1002/ccr3.5158

**Published:** 2021-11-25

**Authors:** Anand Marya, Adith Venugopal

**Affiliations:** ^1^ Department of Orthodontics Faculty of Dentistry University of Puthisastra Phnom Penh Cambodia; ^2^ Department of Orthodontics Saveetha Dental college Saveetha Institute of medical and technical sciences Saveetha University Chennai India

**Keywords:** compound, denticles, odontoma, orthodontic treatment, tumors

## Abstract

Odontomas are common occurrences in the oral cavity and can be classified as complex or compound. Erupted peripheral compound odontomas are rare and present in the extra‐osseous soft tissues. In this case, the odontoma led to the impaction of permanent teeth, due to which removal of the lesion was advised.

## Graphical abstract

Odontomas must be removed in time as these can grow in size and eventually cause destruction of the periodontium, tooth mobility, and damage to adjacent structures. Peripheral compound odontomas must be removed as these have a tendency to exfoliate, and during removal, the area must be inspected for any underlying osseous lesions.

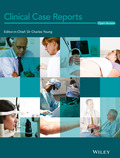

## 1 CASE PRESENTATION

**FIGURE 1:**
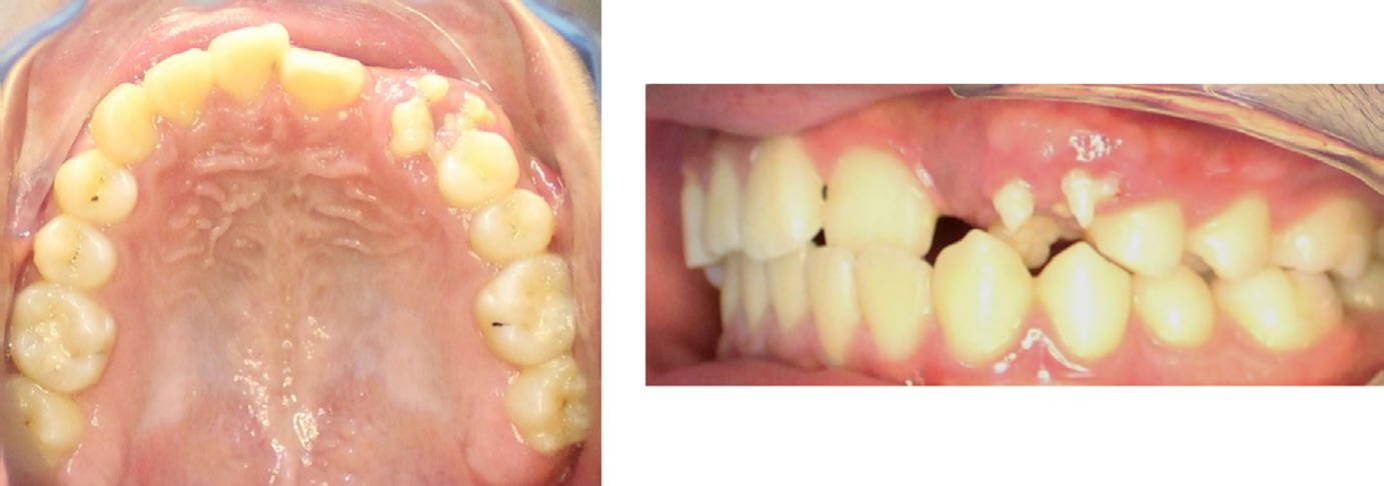
Clinical intra‐oral presentation of the erupted peripheral compound odontoma

**FIGURE 2:**
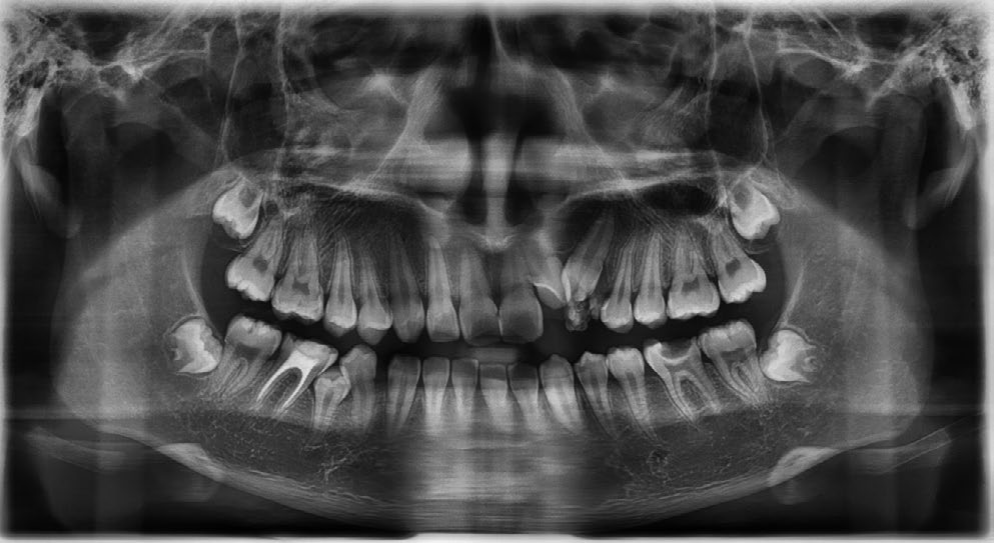
Pre‐treatment panoramic image of the peripheral compound odontoma

**FIGURE 3:**
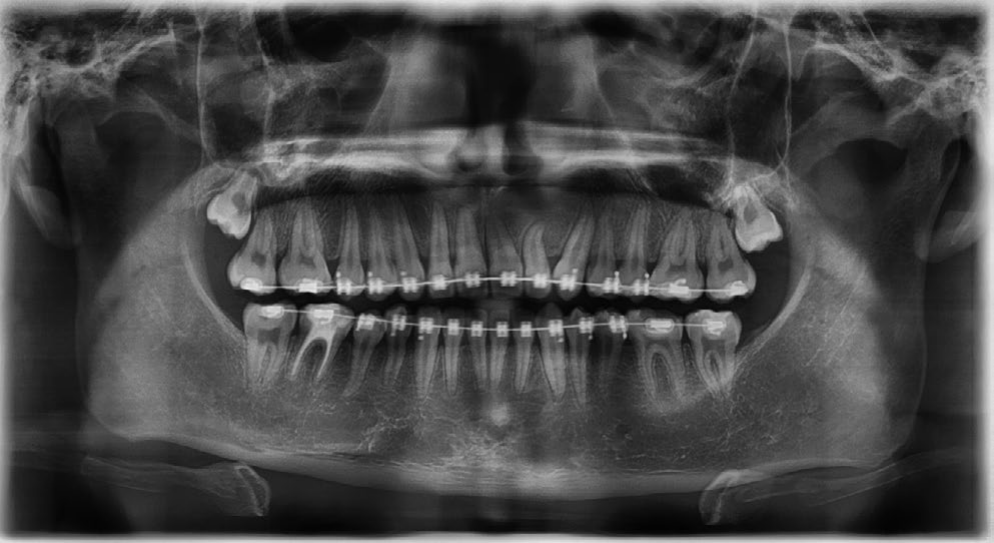
Post‐removal panoramic image of the odontoma with the subsequent alignment of the impacted teeth

Peripheral compound odontomas are considered rare and classified as benign calcified odontogenic tumors.^1^ Eruption of these rare extra‐osseous odontomas may lead to impaction of adjacent teeth.^1^ In this case, a 17‐year‐old boy reported to the university clinic with a problem of missing teeth. On intra‐oral examination, the patient had a missing left lateral incisor and canine, and “Denticles” were present on the gingiva in the affected area^2^ (Figure 1). The patient was asymptomatic on palpation, and the denticles demonstrated biofilm and gingival inflammation around them. Radiographic analysis demonstrated the presence of various irregular tooth structures made up of a crown and the root without bony involvement (Figure 2). The permanent left maxillary lateral incisor and canine were impacted due to their eruptive path being obstructed. Based on these findings, the case was diagnosed as that of peripheral compound odontoma. Since the patient was looking for orthodontic treatment, the patient was advised to remove the odontoma and undergo fixed appliance therapy. Radiologic examination after the alignment of the affected teeth revealed that the lateral incisor root was dilacerated, requiring careful management after that (Figure 3). Dentists must be aware of such problems as these, if not removed, will gradually increase in size and lead to other problems such as adjacent tooth mobility, periodontal destruction, and even esthetic concerns.

## CONFLICT OF INTEREST

The authors made no disclosures.

## AUTHOR CONTRIBUTIONS

AM involved in patient treatment and manuscript preparation. AM and AV involved in diagnosis and treatment planning, and review and editing.

## ETHICAL APPROVAL

Because this report involves no experiment, ethics approval is waived.

## CONSENT

Written informed consent was obtained from the patient to publish this report in accordance with the journal's patient consent policy.

## Data Availability

Any data related to the case can be provided on reasonable request.
